# Postoperative pain after endodontic treatment with glycolic acid as final irrigant using reciprocating and rotary instrumentation in a noninferiority trial

**DOI:** 10.1038/s41598-025-33397-6

**Published:** 2026-02-19

**Authors:** Paola Serraglio Figueiredo, Yuri Dal Bello, João Paulo De Carli, Matheus Albino Souza, Pedro Henrique Corazza, Juliane Bervian, Haron Pedro Lupatini Presser, Gabriela Oltramari Nery, Kamily Konzen, Maria Eduarda Raber, Doglas Cecchin, Micheline Sandini Trentin

**Affiliations:** Dental School of the University of Passo Fundo, Passo Fundo, RS Brazil

**Keywords:** Postoperative pain, Root canal preparation, Ethylenediaminetetraacetic acid, Glycolic acid, Health care, Signs and symptoms

## Abstract

This randomized clinical trial was designed to evaluate whether glycolic acid (GA) is non-inferior to ethylenediaminetetraacetic acid (EDTA) regarding postoperative pain after endodontic treatment. A total of 240 patients requiring treatment were randomly assigned to four groups according to the irrigant (GA or EDTA) and the instrumentation technique (reciprocating or rotary). Postoperative pain was assessed using the numeric rating scale (NRS, 0–10) at 24 h, 48 h, and 7 days. A non-inferiority margin (Δ = 0.9) was prespecified as the maximum acceptable difference between GA and EDTA. Non-inferiority was concluded if the upper bound of the one-sided 95% confidence interval (equivalent to the two-sided 90% CI) for the difference in means (GA – EDTA) was below Δ. Analgesic intake was also recorded. Postoperative pain peaked at 24 h and significantly decreased over time (*p* < 0.001). At all evaluated time points, the upper bound of the one-sided 95% confidence interval for the difference between GA and EDTA remained below Δ, demonstrating that GA was non-inferior to EDTA. Exploratory two-sided analyses revealed no significant differences in pain intensity between irrigants or instrumentation techniques (*p* > 0.05). Most patients reported absent or mild pain, and 79.2% did not require analgesics, with no significant difference among groups (*p* = 0.616). GA is non-inferior to EDTA in terms of postoperative pain following endodontic treatment, regardless of instrumentation technique. Both irrigants provided similar clinical outcomes, with low analgesic intake and favorable patient-centered results. Trial registration number. Brazilian Clinical Trials Registry (ReBec). Registration number RBR-44q9k6q. (Retrospectively registered: October 17, 2023).

## Introduction

Chelating agents are commonly used to remove the smear layer from dentin surfaces, improving the penetration of irrigants and sealers by clearing the dentinal tubules^1,2^. Among them, ethylenediaminetetraacetic acid (EDTA) has long been recommended due to its demineralizing effect on root canal walls^3^. However, EDTA presents drawbacks such as cytotoxicity to periapical tissues^4,5^, reduced dentin microhardness, potential erosive effects, and limited antimicrobial action^4,5^. Additionally, it is a non-biodegradable pollutant, raising environmental concerns^6^.

In this context, glycolic acid (GA), a biodegradable alpha-hydroxy acid, has emerged as a promising alternative^7–9^. In vitro studies have demonstrated that GA has comparable chelating efficacy to 17% EDTA and a more favorable cytotoxicity profile^5,10,11^. Its organic nature suggests a lower potential for periapical irritation, making it a viable candidate as a final irrigant^11^. However, clinical data evaluating the impact of GA on patient-centered outcomes, such as postoperative pain, remain scarce.

Postoperative pain affects 3% to 58% of patients undergoing endodontic treatment, with multiple contributing factors including preoperative symptoms, periapical status, instrumentation technique, and irrigation protocol^12,13^. While mechanized systems have enhanced procedural efficiency, studies comparing rotary and reciprocating instrumentation show conflicting results regarding their effect on postoperative discomfort^14–16^. Reciprocating systems have been associated with greater debris extrusion^17^, potentially increasing postoperative pain. However, Kherlakian et al. (2016)^14^ and Comparin et al. (2017)^18^ found no difference in pain levels when comparing this two kinematics, whereas Nekoofar et al. (2015)^19^ reported that postoperative pain was significantly lower in patients undergoing canal instrumentation with rotary instruments compared with the reciprocating technique. According to the authors, the absence of coronal enlargement in reciprocating systems may increase debris extrusion and, consequently, postoperative pain.

Despite promising laboratory evidence for GA, its behavior in vivo, particularly its influence on postoperative pain, has not been fully explored. Therefore, investigating its clinical performance is essential to determine its potential as a safer and effective alternative to EDTA.

This trial was designed as a non-inferiority randomized clinical trial to test whether glycolic acid (GA) is not clinically worse than EDTA regarding postoperative pain after endodontic retreatment. The trial also aimed to assess the influence of rotary and reciprocating instrumentation techniques on postoperative pain at 24 h, 48 h, and 7 days, as well as the need for analgesic medication.

The study tested three null hypotheses:

1. GA is not inferior to EDTA regarding postoperative pain intensity at all evaluated time points.
2. Postoperative pain does not differ between reciprocating and rotary instrumentation techniques at the same time points.
3. Postoperative pain perception is not associated with the need for analgesic medication across the evaluated groups.

## Materials and methods

This trial was designed as a non-inferiority randomized clinical trial with two parallel arms (GA vs. EDTA), stratified by instrumentation technique (reciprocating -RE and rotary- Ro). A non-inferiority margin (Δ) of 0.9 on the numeric rating scale (NRS, 0–10) was prespecified as the maximum clinically acceptable difference.

This study was conducted at the School of Dentistry of the University of Passo Fundo, Brazil, from August 2022 to August 2023. It included 240 patients recruited from the pool of patients attending the Department of Endodontics. Table 1 presents the characteristics of the study group. Study preparation followed the CONSORT Statement (www.consort-statment.org)^20^, and it received approval from the Research Ethics Committee of the University of Passo Fundo (No. 5.569.096). It was conducted in accordance with accepted ethical standards for research practice. Date of registration August 08, 2022). All enrolled participants signed a written informed consent. The study protocol was registered in the Brazilian Clinical Trials Registry (ReBec) (October 17, 2023) under the number RBR-44q9k6q.

### Sample size calculation

The sample size was estimated based on a non-inferiority design to compare postoperative pain between GA and EDTA in each instrumentation modality (reciprocating and rotary). The primary outcome was postoperative pain intensity measured on the NRS scale. A non-inferiority margin (Δ) of 0.9 was prespecified as the maximum acceptable difference between groups. Based on preliminary data, a standard deviation of 2.6 was assumed. Using a one-sided significance level of 0.05 and 80% power, the required sample size was 51 participants per group (204 patients in total). To increase power and account for potential dropouts, 240 patients were included.

### Selection of volunteers

Participants of both genders were registered for endodontic treatment at the Department of Endodontics from August 2022 to August 2023. All teeth had undergone endodontic access procedures, provisionally restored at a public dental healthcare service, and referred for definitive treatment. Before initiating the research, the need for endodontic treatment was determined by collecting the participants’ dental history and performing digital periapical radiography, periodontal evaluation, and percussion and cold tests.

### Inclusion criteria

The inclusion criteria were patients of both genders, aged 18 to 80 years, whose teeth had undergone access cavity preparation (previously initiated therapy) in public healthcare service and were subsequently referred to the university for endodontic treatment. Depending on the extent of prior therapy, the tooth might or might not respond to pulp sensitivity tests^21^. All patients underwent a clinical examination that included percussion testing. Vertical percussion was performed on the incisal or occlusal surface, while horizontal percussion was applied to the buccal surface of the tooth. Only teeth that elicited no pain or presented, at most, mild sensitivity upon percussion were included in the study, as the absence of significant painful symptoms at the time of evaluation was a necessary condition for inclusion.

### Exclusion criteria

The exclusion criteria comprised patients with systemic conditions, such as diabetes mellitus, hypertension, osteoporosis, or heart disease, pregnant or lactating women, and those using analgesics. Patients who did not consent to participate or discontinued treatment were also excluded from the study.

The study did not include teeth indicated for endodontic retreatment, with root resorption, immature or open apices, or filling material extrusion beyond the apical foramen. Root canals that did not achieve foraminal patency were also excluded.

Before treatment, all teeth were evaluated using periapical radiographs taken from multiple angulations. Only teeth with a Periapical Index (PAI) score between 1 and 3 were included, ensuring the exclusion of cases with advanced apical periodontitis while allowing the inclusion of teeth with normal periapical structures or minor radiographic changes. When necessary, cone-beam computed tomography (CBCT) was used to assess root and canal morphology.

Teeth were included only if their canals could be clearly identified, negotiated, instrumented, and filled. Teeth with anatomical anomalies—such as C-shaped canals, severe curvatures, internal resorptions, or severely calcified canals—were excluded. In cases where untreated canals were suspected on the final radiograph, a CBCT scan was performed for confirmation. If the suspicion was verified, the tooth was excluded from the study to minimize anatomical variability and avoid bias related to incomplete canal preparation.

### Random selection

A total of 240 patients who had undergone partial endodontic therapy were recruited for treatment. The allocation method applied block randomization. After selecting the volunteers and before starting treatment, the researcher randomly selected the instrumentation system by drawing a card from a brown envelope. This envelope contained 20 cards labeled “reciprocating” and 20 cards labeled “rotatory,” determining the instrumentation system. The same randomization method defined the final irrigating solution, with options for GA or EDTA. Although the operator could not be blinded to the instrumentation kinematics, both the operator and the patient were blinded to the irrigating solution, which was provided in identical syringes. Thus, the study followed a single-blind design.

### Treatment protocol

Postgraduate endodontic students with the same training and experience at the time of the study performed the treatments.

Local anesthesia was administered by infiltrating 3.6 mL of 2% lidocaine with 1:100,000 epinephrine (Alphacaine; DFL Indústria e Comércio Ltda, Rio de Janeiro, RJ, Brazil). The endodontic access employed 1012 diamond and Endo-Z burs (Dentsply Maillefer, Ballaigues, Switzerland) under rubber dam isolation, followed by canal exploration with a #10 K-file (Dentsply Sirona, Ballaigues, Switzerland).

Canal instrumentation was performed according to the randomized allocation to each group: Reciproc (VDW, Munich, Germany) or Wave One Gold (Dentsply Sirona, Switzerland) for reciprocating kinematics (RE) and ProTaper Gold (Dentsply Tulsa, Johnson City, TN) or ProT (MkLife, Munich, Germany) for rotatory kinematics (RO). An X-Smart Plus electric motor (Dentsply Maillefer, Ballaigues, Switzerland) was used, and all instruments were operated according to the manufacturers’ predefined instructions.

Regarding the reciprocating movement, the preselected file aided the preparation of two -thirds of the canal length before determining the working length (WL). In rotational kinematics, the SX file provided cervical flaring, followed by the S1 file until reaching two-thirds of the canal. The WL was then established by introducing a #10 or #15 K-file to the apical foramen using an electronic apex locator (Dentsply Sirona, Switzerland) at the “0.0” point.

All root canals were instrumented with 2% chlorhexidine gel (Natupharma Manipulação & Medicamentos, Passo Fundo, RS, Brazil) as a lubricant, which was subsequently removed with 5 mL of saline solution (NaviTip Tips 30G, Ultradent Products Inc., South Jordan, UT, USA) at each instrument change. The irrigation needle was inserted 3 mm short of the WL.

The smear layer removal protocol involved 5 mL of the test solution (GA or EDTA), delivered along the entire length of the root canal. Passive ultrasonic irrigation (PUI) with an E1 tip (Irrisonic, Helse Ultrasonic, Ribeirão Preto, SP, Brazil) attached to a Satelec ultrasonic device (Acteon Group, Merignac, France) agitated the solution at 20% intensity. The final irrigating solution was activated in three 20-second cycles, renewing the solution after each cycle.

The root canals were subsequently irrigated with 0.9% saline solution and dried using sterile absorbent paper points (Dentsply Sirona Endodontics, Switzerland). The canals were then filled using the single-cone gutta-percha technique (Odous; Odous De Deus Ltd, Belo Horizonte, Brazil) combined with Sealer Plus endodontic cement (MkLife, Munich, Germany). A final digital periapical radiograph was taken, excluding any teeth with iatrogenic alterations (e.g., perforations, underfilling, or overfilling) from the study.

All the access cavities underwent permanent restoration with a flowable resin layer (Tetric, Ivoclar Vivadent AG, Schaan, Liechtenstein) followed by composite resin layers (Z250, 3 M ESPE, Sumaré, SP, Brazil) applied using the incremental technique with an adhesive system (Single Bond, 3 M ESPE, Sumaré, SP, Brazil). Occlusal adjustments were performed carefully to ensure proper function.

After endodontic treatment, all patients received postoperative instructions, including the recommendation to use analgesics if necessary. They were advised to take a 300 mg tablet of ibuprofen (Laboratory Teuto, Minas Gerais, Brazil), with subsequent doses at six-hour intervals, in case one tablet was insufficient to relieve the pain.

### Postoperative pain analysis

The participants received a questionnaire based on a numerical rating scale (NRS) to record their pain assessment at 24 h, 48 h, and 7 days post-treatment. The scale consists of a continuous horizontal line ranging from 1 to 10, with the numerical values grouped into visual categories. The patients were instructed to assign a value corresponding to their perceived pain level using this scale.

The blinded researcher-evaluator contacted the participants by phone at scheduled intervals of 24 h, 48 h, and 7 days after treatment to monitor postoperative pain, complete the verbal description scale, and ask the following questions: “What is your current pain perception?” and “Did you need to use the prescribed medication? Yes or no?” All participants were instructed to contact the researcher responsible for their treatment if the prescribed analgesic did not relieve pain or any other complication occurred.

### Statistical analysis

The statistical analyses used SPSS software (Statistical Package for the Social Sciences, v. 22.0; IBM Corp, Chicago, IL). The primary analysis tested the non-inferiority of GA compared with EDTA regarding mean postoperative pain at each time point. Non-inferiority was concluded if the upper bound of the one-sided 95% confidence interval (equivalent to a two-sided 90% CI) for the difference in means (GA – EDTA) was below the non-inferiority margin (Δ = 0.9).

Exploratory two-sided tests were also performed to compare groups beyond the non-inferiority framework. Pain intensity over time was analyzed with repeated-measures models. A significance level of *p* < 0.05 was adopted for exploratory two-sided analyses.

**Fig. 1:**
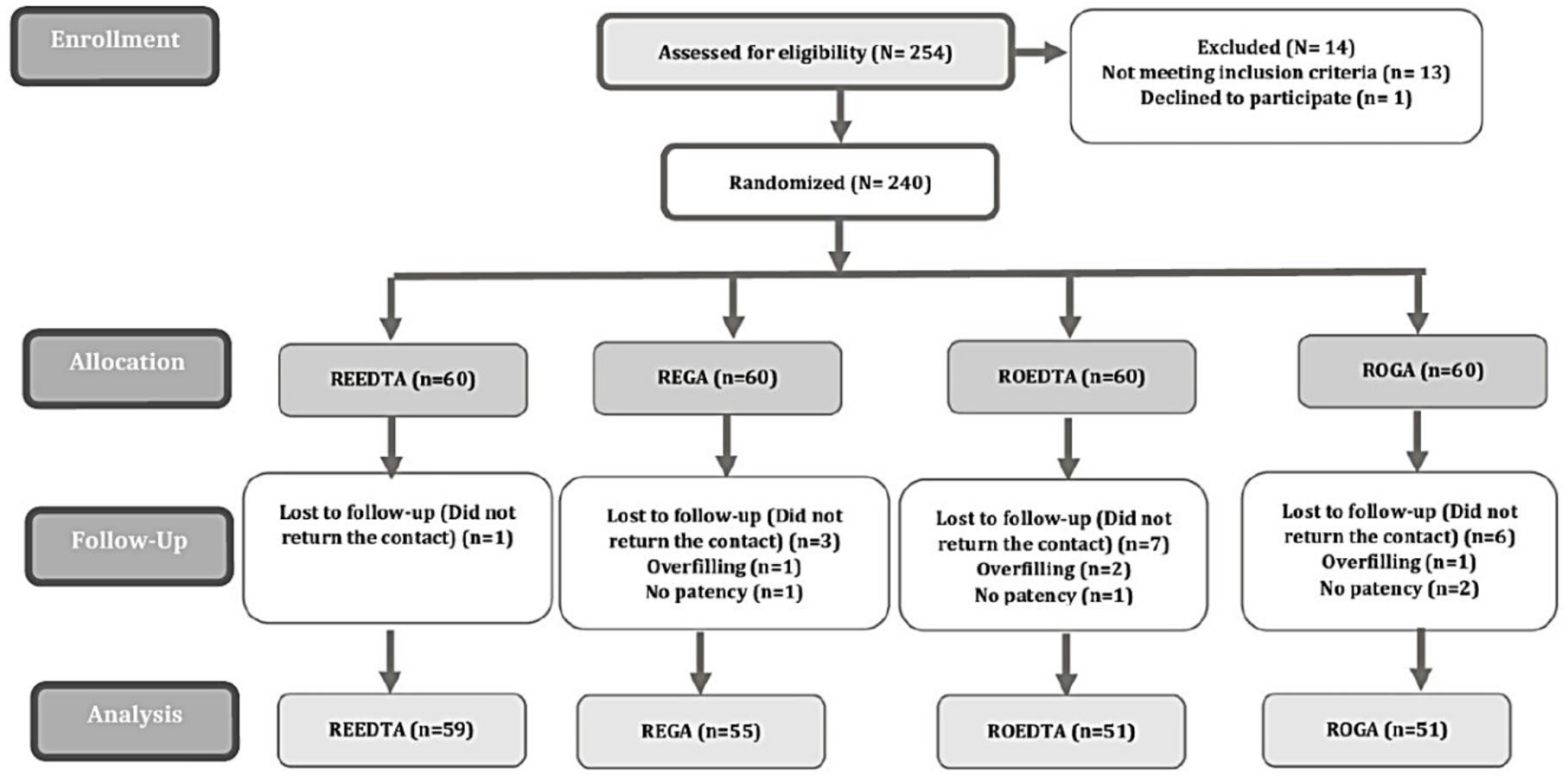
CONSORT flow chart of the study.

## Results

Figure 1 presents the study flowchart. Among the 254 patients initially selected, 240 were scheduled for treatment. After dropouts, 216 patients were treated and evaluated between August 2022 and August 2023. The retention rate was approximately 90%, as 24 patients (10%) were excluded due to the lack of follow-up, overfilling, or failure to achieve patency (Fig. 1).

Table 1 shows the baseline demographics and clinical characteristics of the study groups. The mean age of participants was 43.9 years. As for gender, 66.2% (143) were women and 33.8% (73) were men. Among the 216 treated teeth, 57% (123) were in the maxilla and 43% (93) in the mandible. Anterior teeth represented 19.4% (42), whereas 80.6% (174) were premolars and molars.

Postoperative pain was highest at 24 h and decreased significantly over time, reaching minimal levels at 7 days (*p* < 0.001 for time trend) (Table 2).

In the primary non-inferiority analysis, the upper bound of the one-sided 95% confidence interval for the difference in mean postoperative pain (GA – EDTA) at all evaluated time points was below the non-inferiority margin (Δ = 0.9). Therefore, GA was demonstrated to be non-inferior to EDTA for postoperative pain control after treatment. In exploratory two-sided comparisons, no statistically significant differences in mean postoperative pain were detected between irrigants or instrumentation techniques (*p* > 0.05).

Pairwise comparisons using the Mann–Whitney test revealed no statistically significant differences in pain levels between rotary (RO) and reciprocating (RE) instrumentation systems, nor between glycolic acid (GA) and ethylenediaminetetraacetic acid (EDTA) as final irrigants, at any evaluated time point (*p* > 0.05). The rank-biserial correlations were small, further indicating negligible effect sizes among groups.

At 48 h, pain scores remained low, with median values of 0.0 and 95% CIs ranging from 0.21 to 2.16. Again, no significant intergroup differences were detected (*p* > 0.05). By 7 days postoperatively, pain had largely resolved in all groups, with median scores of 0.0 and narrow CIs close to zero. In particular, the ROGA group showed complete absence of pain, with zero variance at this time point.

**Table 1:** Baseline demographics and clinical characteristics of each group (*n* = 216).

Characteristic	Category	Group			
		REEDTA (*n* = 59)	REGA (*n* = 55)	ROEDTA (*n* = 51)	ROAG (*n* = 51)
Age – years Mean (SD)	-	43.69 (15.8)	40.56 (16.5)	42.8 (14.4)	47.59 (16.9)
Gender (%)	Female	33 (55.9%)	41 (74.5%)	39 (76.5%)	30 (58.8%)
	Male	26 (44.1%)	14 (25.5%)	12 (23.5%)	21 (41.2%)
Tooth position (%)	Maxillary arch	37 (62.7%)	29 (52.7%)	29 (56.9%)	28 (54.9%)
	Mandibular arch	22 (37.3%)	26 (47.3%)	22 (43.1%)	23 (45.1%)
Tooth (%)	Incisor/canine	14 (23.7%)	9 (16.4%)	10 (19.6%)	9 (17.6%)
	Premolar	20 (33.9%)	9 (16.4%)	12 (23.5%)	19 (37.3%)
	Molar	25 (42.4%)	37 (67.3%)	29 (56.9%)	23 (41.5%)
Diagnosis (%)	Previously initiated therapy	59 (100%)	55 (100%)	51 (100%)	51 (100%)
Instrumentation Technique (%)	Rotatory	–	–	51 (100%)	51 (100%)
	Reciprocating	59 (100%)	55 (100%)	–	–
Final irrigation (%)	EDTA	59 (100%)	–	51 (100%)	–
	Glycolic acid	–	55 (100%)	–	51 (100%)
PUI (%)	Irrisonic E1	59 (100%)	55 (100%)	51 (100%)	51 (100%)
Cementation (%)	Single cone	59 (100%)	55 (100%)	51 (100%)	51 (100%)
Number of sessions (%)	One visit	35 (59.3%)	27 (49.1%)	18 (35.3%)	31 (60.8%)
	Multiple visits	24 (40.7%)	28 (50.9%)	33 (64.7%)	20 (39.2%)
Postoperative medication (%)	Yes	10 (16.9%)	14 (25.5%)	12 (23.5%)	9 (17.6%)
	No	49 (83.1%)	41 (74.5%)	39 (76.5%)	42 (82.4%)

**Table 2:** Descriptive statistics and Mann–Whitney test applied to each experimental group within their postoperative pain.

Pain assessment	Group	*n*	Median (95% CI)	IQR^&^	Rank-biserial correlation	*p*-value*
Pain after 24 h	REEDTA	59	0.0 (1.00–2.34)	2.5	0.108	0.164
	REGA	55	0.0 (1.38–3.05)	3.5		
	ROEDTA	51	0.0 (1.07–2.69)	3.0	0.114	0.850
	ROGA	51	0.0 (0.60–1.98)	1.5		
Pain after 48 h	REEDTA	59	0.0 (0.73–1.94)	2.0	0.108	0.565
	REGA	55	0.0 (0.71–2.16)	2.0		
	ROEDTA	51	0.0 (0.54–1.88)	1.5	0.114	0.887
	ROGA	51	0.0 (0.21–1.20)	0.0		
Pain after 7 days	REEDTA	59	0.0 (0.00–0.66)	0.0	0.108	0.586
	REGA	55	0.0 (0.00–0.68)	0.0		
	ROEDTA	51	0.0 (0.00–0.93)	0.0	0.0^a^	0.0^a^
	ROGA	51	0.0 (0.00–0.00)	0.0		

*Mann-Whitney test (*p* > 0.05).

**For the Mann-Whitney test, effect size is given by the rank biserial correlation.

***For all tests, the alternative hypothesis specifies that group *EDTA* is less than group *GA*.

^a^The variance in Pain 7 days is equal to 0 after grouping on Irrigant.

^&^Interquartile range (IQR).

Within-group analysis of postoperative pain over time (Table 3) demonstrated a consistent and significant reduction from 24 h to 7 days (*p* < 0.001), despite median pain scores remaining at 0.0 throughout.

In the REEDTA group, the median pain score between 24 and 48 h was 0.0 (95% CI: 1.06–1.95; IQR = 2.0), with no significant difference (*p* = 0.569). Significant reductions were observed between 24 h and 7 days (*p* < 0.001), and between 48 h and 7 days (*p* < 0.001), with IQR narrowing to 0.0, indicating marked pain relief by day 7. REGA group, pain decreased significantly between 24 and 48 h (*p* = 0.04), suggesting early improvement. Further significant reduction occurred between 24 h and 7 days (*p* < 0.001), while no difference was observed between 48 h and 7 days (*p* = 0.183).

For the ROEDTA group, no significant change was observed between 24 and 48 h (*p* = 0.222). However, pain decreased significantly between 24 h and 7 days (*p* < 0.001) and between 48 h and 7 days (*p* = 0.005).

**Table 3:** Descriptive statistics and the Kruskal-Wallis test applied to each group in their pain ranges:

Group	Time	*n*	Median (95% CI)	Min - Max	IQR	*p*-value*
REEDTA	24hx48h	59	0.0 (1.06 to 1.95)	0–8	2.0	0.569
	24hx7days	59	0.0 (0.60 to 1.39)	0–8	0.0	< 0.001
	48hx7 days	59	0.0 (0.47 to 1.18)	0–8	0.0	< 0.001
REGA	24hx48h	55	0.0 (1.19 to 2.58)	0–10	3.0	0.04
	24hx7days	55	0.0 (0.80 to 1.75)	0–10	1.0	< 0.001
	48hx7 days	55	0.0 (0.10 to 0.89)	0–10	0.0	0.183
ROEDTA	24hx48h	51	0.0 (1.02 to 2.06)	0–10	2.0	0.222
	24hx7days	51	0.0 (0.68 to 1.65)	0–10	0.0	< 0.001
	48hx7 days	51	0.0 (0.42 to 1.24)	0–10	0.0	0.005
ROGA	24hx48h	51	0.0 (0.57 to 1.42)	0–10	1.0	0.164
	24hx7days	51	0.0 (0.28 to 1.01)	0–8	0.0	0.00^a^
	48hx7 days	51	0.0 (0.10 to 0.60)	0–0	0.0	0.00^a^

*Mann-Whitney test *p* < 0.05.

^a^ The variance in pain is equal to 0.

In the ROGA group, no significant difference was found between 24 and 48 h (*p* = 0.164). Nevertheless, significant reductions were observed between 24 h and 7 days, and between 48 h and 7 days (both *p* < 0.001). Notably, variance in pain scores at 7 days was zero, confirming complete pain resolution in all participants.

Pain scores across groups and time points ranged from 0 to a maximum of 8–10, with IQRs progressively decreasing to 0.0, supporting the consistent resolution of postoperative pain within 7 days.

Regarding pain intensity categories (Table 4), no/mild pain was the most common across all groups, ranging from 78.8% to 89.5%. Moderate pain occurred in 7.8% to 15.3% of cases, whereas severe pain was the least frequent category, consistently below 8% in all groups.

Postoperative medication use (Table 5) was reported by 20.8% (45) of cases, whereas 79.2% (171) of treated teeth required no analgesics. The highest medication usage rates were observed in the REGA (25.5%) and ROEDTA (23.5%) groups, though no statistically significant differences in medication use were found among the groups (*p* = 0.616).

**Table 4:** Frequencies of pain categories: no/mild pain (level 1 – scores 0 to 2), moderate pain (level 2 – scores 3 to 7), and severe pain (level 3 – scores 8 to 10).

Group	Pain	Frequency	Percent
REGA	Moderate	23	13.9
	No/mild	130	78.8
	Severe	12	7.3
	Total	165	100.0
REEDTA	Moderate	27	15.3
	No/mild	147	83.1
	Severe	3	1.7
	Total	177	100.0
ROEDTA	Moderate	23	15.0
	No/mild	124	81.0
	Severe	6	3.9
	Total	153	100.0
ROGA	Moderate	12	7.8
	No/mild	137	89.5
	Severe	4	2.6
	Total	153	100.0

**Table 5:** Descriptive statistics and the Kruskal-Wallis test applied to the groups and postoperative medication use.

Group	Postoperative medication		Total	*p*-value*
	No (%)	Yes (%)		
REEDTA	49 (83.1)	10 (16.9)	59 (100)	0.616
REGA	41 (74.5)	14 (25.5)	55 (100)	
ROEDTA	39 (76.5)	12 (23.5)	51 (100)	
ROGA	42 (82.4)	9 (17.6)	51 (100)	
Total	171 (79.2)	45 (20.8)	216 (100)	

*Kruskal Wallis test *p* > 0.05.

Postoperative pain and the number of sessions (single vs. multiple) were not significantly different at 24 h (*p* = 0.190), 48 h (*p* = 0.455), or 7 days (*p* = 0.459). Similarly, pain levels did not differ significantly between arches (maxilla vs. mandible) at 24 h (*p* = 0.257), 48 h (*p* = 0.240), or 7 days (*p* = 0.173). The results were comparable regarding gender (male vs. female) at 24 h (*p* = 0.817), 48 h (*p* = 0.977), and 7 days (*p* = 0.291).

## Discussion

This randomized clinical trial demonstrated that GA is non-inferior to EDTA in terms of postoperative pain after endodontic treatment. The prespecified non-inferiority margin (Δ = 0.9 on the NRS scale) was not exceeded at any time point, confirming that GA provides postoperative outcomes that are clinically comparable to those of EDTA. The present study found no statistically significant differences in pain perception between the evaluated instrumentation kinematics (rotary or reciprocating) or final irrigating solutions (GA or EDTA), confirming the first and second study hypotheses.

One of the main challenges in assessing postoperative pain in clinical studies is its subjective nature and the inherent difficulty of accurately measuring it^14^. The numerical rating scale (NRS) was employed in this study, as it is widely recognized for pain assessment due to its reliability, cross-cultural adaptability, and superior sensitivity compared with other methods^19,22^. In addition, the NRS allows patients to independently report their pain levels, minimizing potential biases introduced by researchers or operators^22,23^.

Preoperative pain is a strong predictor of postoperative pain^24^. Therefore, all participants in this study were required to report no spontaneous pain at the start of treatment to minimize bias. Mild discomfort in response to percussion—whether vertical or horizontal—was accepted, provided it did not compromise the standardization of clinical conditions. This strategy enabled the inclusion of cases with minimal periapical inflammation, while still maintaining homogeneity in the study sample. Postoperative pain was highest within the first 24–48 h, with a significant reduction by day 7. This finding corroborates previous studies that reported higher pain incidence within 24 h, regardless of the instrumentation system used^14,18,25,26^.

To minimize variability in pain perception caused by anatomical complexity or incomplete instrumentation, teeth with suspected untreated canals on the final radiograph were further examined using CBCT. If untreated canals were confirmed, the cases were excluded. While this strict criterion enhanced the internal validity of the study, it may limit generalizability, as it does not fully reflect the heterogeneity of cases typically encountered in routine clinical practice.

Across the 216 treated teeth, postoperative pain was most frequently categorized as “no/mild pain,” with prevalence rates of 72.2% at 24 h, 81.5% at 48 h, and 95.4% at 7 days. Moderate pain ranged from 20.8% (24 h) to 4.2% (7 days), and severe pain was rare, decreasing from 6.9% (24 h) to only 0.5% at 7 days. Kherlakian et al.^14^ and Comparin et al.^18^ reported similar findings, with postoperative pain affecting 25–40% of patients, as did Sathorn et al.,^27^ who reported pain prevalence rates from 3% to 58%.

Although Lago et al.^28^ reported significantly lower pain levels with GA compared to EDTA within the first 24 h, the present study did not confirm these differences. Interestingly, the lowest pain scores were observed in the ROGA group, suggesting that postoperative pain is likely influenced by a combination of factors, including both the chelating agent and the instrumentation kinematics, rather than by the irrigant alone.

Reciprocating systems have been associated with greater debris extrusion^17^, potentially increasing postoperative pain. However, in this study, no significant differences were found between reciprocating and rotary systems. Similar findings have been reported in other clinical studies with no difference between two reciprocating and one rotatory system^14^. Nonetheless, Nekoofar et al.^19^ presented contrasting findings, including significantly lower postoperative pain in patients treated with rotary systems. The authors attributed this discrepancy possibly to differences in inclusion criteria (e.g., diagnosis of irreversible pulpitis vs. vital teeth), irrigating solutions (chlorhexidine vs. NaOCl), and kinematic protocols for reciprocating instrumentation^14^. The operators of the present study strictly followed the manufacturers’ instructions for instrumentation.

Although sodium hypochlorite (NaOCl) remains the gold standard in endodontics due to its tissue-dissolving and antimicrobial properties, chlorhexidine was selected as an auxiliary irrigant in this study due to its broad antimicrobial spectrum, residual activity, lower cytotoxicity, and favorable clinical performance. Its gel formulation provides additional lubricating and rheological benefits, and it is chemically stable and water-soluble^29^. Previous clinical studies have reported similar clinical outcomes between NaOCl and chlorhexidine^30–32^ and a recent meta-analysis concluded that both irrigants are equally effective despite their distinct mechanisms of action^33^. Therefore, EDTA was used as the control irrigant in this study, as chlorhexidine was consistently employed as an auxiliary substance across all experimental groups.

In vitro studies^5,11^ have demonstrated that GA exhibits lower cytotoxicity and comparable smear layer removal to EDTA, with less dentin erosion^10^. EDTA strongly chelates calcium, an essential ion for cell function^34^, whereas GA—due to its low molecular weight and organic nature^5^ —primarily acts through its acidity and penetrative capacity^35^. The greater calcium depletion caused by EDTA may explain its higher cytotoxic potential and possible association with a stronger inflammatory response. Despite these biological differences, this clinical trial did not find significant differences in postoperative pain between GA and EDTA, corroborating previous clinical study^28^.

Patient-related variables such as age, gender, tooth type, and number of sessions have been linked to postoperative pain in prior studies^18,36,37^. In the present trial, pain outcomes were not significantly influenced by these factors. Although gender distribution was not homogeneous across groups, postoperative pain did not differ significantly between men and women, suggesting that this imbalance likely did not affect the results.

Previous studies have identified associations between increased pain and factors such as older age, mandibular teeth^38^, and gender, with women being more frequently affected^39^. However, conflicting results have been reported. Comparin et al.^18^ detected a higher pain incidence in men within 24 h, while Sadaf and Ahmad^36^ found more frequent pain in women (65% vs. 35%). The present study did not find associations between postoperative pain and variables such as gender, dental arch, or the number of sessions (*p* > 0.05). These findings may be attributed to the use of a standardized treatment protocol and rigorous inclusion criteria.

Analgesic use was similar across groups, supporting the third study hypothesis. Most patients used analgesics for approximately two days, with occasional extended use up to five days in cases of exacerbated pain. NSAIDs were the first-line medication, consistent with current recommendations^40^. Importantly, these medications were provided by the public healthcare system, ensuring accessibility for all participants.

No significant differences were found in postoperative pain between single- and multiple-session treatments. Schwendicke and Göstemeyer^37^ showed similar findings, stating that the risk of postoperative complications was comparable between single and multiple sessions of endodontic treatment. However, the risk of flare-ups following a single session was significantly higher than that observed after multiple sessions.

This study has limitations. Treatments were performed by multiple operators, which may have introduced variability, although all procedures followed standardized protocols. Patient-related variables were not randomized, which may have affected external validity. Analgesic intake was recorded only as a binary variable (yes/no), without quantifying dosage or frequency, limiting the accuracy of medication-related findings. Future studies should consider recording the precise number and type of analgesic doses to allow a more robust evaluation of postoperative medication needs. In addition, further research is required to assess the long-term outcomes of GA, including its effects on apical healing and clinical performance in cases with preoperative pain.

## Conclusion

GA was non-inferior to EDTA in terms of postoperative pain after endodontic treatment, suggesting that GA may represent a viable alternative as a final chelating solution. Nevertheless, further clinical trials with larger samples and longer follow-up are needed to confirm these findings.

## Data Availability

The datasets used and analyzed during the current study are available from the corresponding author upon reasonable request.
